# Antibacterial Synergism of Electrospun Nanofiber Mats Functioned with Silver Nanoparticles and Pulsed Electromagnetic Waves

**DOI:** 10.3390/polym13020277

**Published:** 2021-01-15

**Authors:** Mai I. El-kaliuoby, Alaa M. Khalil, Ahmed M. El-Khatib, Nader Shehata

**Affiliations:** 1 Faculty of Education, Alexandria University, Alexandria 21544, Egypt; mai.ismail@alexu.edu.eg; 2 Faculty of Engineering, Pharos University in Alexandria, Alexandria 21544, Egypt; alaa.khalil@pua.edu.eg; 3 Faculty of Science, Alexandria University, Alexandria 21544, Egypt; elkhatib60@yahoo.com; 4 Department of Engineering Mathematics and Physics, Faculty of Engineering, Alexandria University, Alexandria 21544, Egypt; 5 Center of Smart Materials, Nanotechnology, and Photonics (CSMNP), Smart Critical Infrastructure (SmartCI) Research Center, Alexandria University, Alexandria 21544, Egypt; 6 Kuwait College of Science and Technology, Doha Area, 7th Ring Road, Safat 13133, Kuwait; 7 USTAR Bio-Innovation Center, Utah State University, Logan, UT 84341, USA

**Keywords:** nanofibers, silver nanoparticles, electrospinning, electromagnetic waves, antibacterial *E. coli*, *S. aureus*

## Abstract

The over-reliance on antibiotics and their enormous misuse has led to warnings of a future without effective medicines and so, the need for alternatives to antibiotics has become a must. Non-traditional antibacterial treatment was performed by using an aray of nanocomposites synergised with exposure to electromagnetic waves. In this manuscript, electrospun poly(vinyl alcohol) (PVA) nanofiber mats embedded with silver nanoparticles (Ag NPs) were synthesized. The nanocomposites were characterized by Transmission Electron Microscope (TEM), Scanning Electron Microscope (SEM), Current-Voltage (I-V) curves, and Thermogravimetric analysis (TGA) along with analysis of antibacterial impact against *E. coli* and *S. aureus* bacteria, studied by bacterial growing analysis, growth kinetics, and cellular cytotoxicity. The results indicated a spherical grain shape of silver of average size 20 nm and nanofibers’ mean diameter of less than 100 nm. The nanocomposite mats showed good exposure to bacteria and the ability to sustain release of silver for a relatively long time. Moreover, the applied electromagnetic waves (EMWs) were shown to be a synergistic co-factor in killing bacteria even at low concentrations of Ag NPs. This caused pronounced alterations of the bacterial preserved packing of the cell membrane. Thereby, the treatment with nanocomposite mats under EM wave exposure elucidated maximum inhibition for both bacterial strains. It was concluded that the functioning of nanofiber with silver nanoparticles and exposure to electromagnetic waves improved the antibacterial impact compared to each one alone.

## 1. Introduction

The textile industry in the medical and healthcare direction nowadays is growing at a fast pace [1]. The severe exposure to different viruses, bacteria, and fungi in normal life routinely leads to remarkable deteriorative human health consequences. On the other hand, the widespread use of antibiotics has resulted in many bacterial strains becoming highly resistant. Therefore, the need for safe procedures and precautions to prevent infection rather than treatment after infection has become a must. Many alternatives to antibiotics could be achieved by using the advantage of nanotechnology which has particularly high impact on medical and surgical applications. In this regard, silver nanoparticles (Ag NPs) have a great effect in overcoming bacteria in dental therapies, catheters, burns, wounds, and other examples [2]. Silver ions (Ag^+^) have the ability to cross biological membranes [3] and cause remarkable bactericidal impact on divergent types of bacterial strains. Moreover, these metallic ions are able to intercalate between purine and pyrimidine base pairs disrupting the hydrogen bonding between the two anti-parallel strands and denaturing the DNA molecule [4]. Further, Ag atoms can bind to thiol groups (–SH) in enzymes and subsequently cause enzyme deactivation [5]. Its antibacterial action comes from surface oxidation by the release of Ag^+^ ions, generation of reactive oxygen species (ROS), and direct contact with the cellular membrane [6,7,8]. On the contrary, the high dose of Ag NPs induces a toxic response to different mammalian cell lines [9]. So, one may use an alternative combination to get maximum antibacterial effectivity in using Ag NPs at low concentrations [10]. Several research works were instigated to combine various types of antibiotics with Ag NPs and the obtained data showed better synergism between combination compared to one alone [11]. In a further direction, the combination between newly manufactured materials and nanoparticles, to form nanocomposites such as nanofibers loaded in-situ with nanoparticles, showed a remarkable antibacterial effect. In this regard, one of the most recent promising techniques for preparing polymer nanofibers is electrospinning. The electrospun nanofibers (ENFs) are considered as simple, effective, relatively low cost, and have the ability to produce nanomaterials encapsulated inside nanofibers. These impregnated nanofiber mats have unique properties in improving effectivity in topical drug administration and wound healing [12,13]. Particularly, such nanocomposites have higher surface area-to-volume ratio, higher porosity, and smaller pore sizes that allow the nanoparticles to be well dispersed [14,15]. In this regard, ultrafine poly(acrylonitrile) (PAN) fibers containing and ultra-fine cellulose acetate (CA) fibers containing Ag NPs were prepared by using electrospinning for antibacterial medical applications [16]. The obtained results indicated that low concentrations of Ag NPs were needed to obtain clear effective antibacterial properties. One of the most promising organic hosts for metals is poly (vinyl alcohol) (PVA), due to its premium thermo-stability, biocompatibility, and chemical resistance [17,18]. Moreover, PVA containing Ag NPs showed good hydrophilicity, biocompatibility, and antimicrobial effect as reported by Shao, C. et al. [19]. Indeed, the hybrid loading of Ag NPs to PVA as a vehicle polymeric matrix plays a crucial role in improving physical, chemical, and mechanical properties of polymers and antibacterial properties of nanoparticles [20]. Lately, another example has been demonstrated using electromagnetic waves (EMWs) as cofactor to enhance the antibacterial activity of Ag NPs [21]. The obtained results indicated significant synergistic combination of very low concentrations of Ag NPs with EMWs on the basis of size and shape. So, the former findings give us insights into using such EMWs as co-stressor factor against bacteria in combination with nanomaterials. 

In this work, PVA nanofibers loaded with Ag NPs were synthesized by the electrospinning method and then the generated nanofibers mat was adopted to study its antibacterial synergistic impact in combination with exposure to EMWs. Our synthesized silver-embedded-PVA nanofibers mat was analyzed using both thin-film conductivity and thermogravimetric Analysis (TGA) to prove the existence of incremented silver nanoparticles either inside or on the morphological surface of the nanofibers. Furthermore, the bacterial growth characteristics, growing dynamics, and cell cytotoxicity were examined in investigating the treatment effects on gram positive and negative bacterial strains. 

## 2. Materials and Methods

### 2.1. Materials

The silver nanoparticles (Ag NPs) were synthesized by using the arc discharge method mechanically driven by a rotational disk electrode automated by a micro-electronic system as reported by El-khatib et al. [19], and collected in deionized water. A concentration of 10 wt% poly(vinyl alcohol) (PVA) solution was prepared by mixing 10 g PVA pellets (Mowoil-10-98 Sigma Aldrich, St. Louis, MO, USA) with 90 mL of distilled water. The solution was heated to 100 °C for 30 min then stirred overnight. This weight concentration was experimentally found to be optimum for generation nanofibers mats with no beads. Silver nanoparticles were mixed in-situ with PVA solution and stirred for 30 min before entering the electrospinning process. 

### 2.2. Electrospinning Process

**Figure 1:**
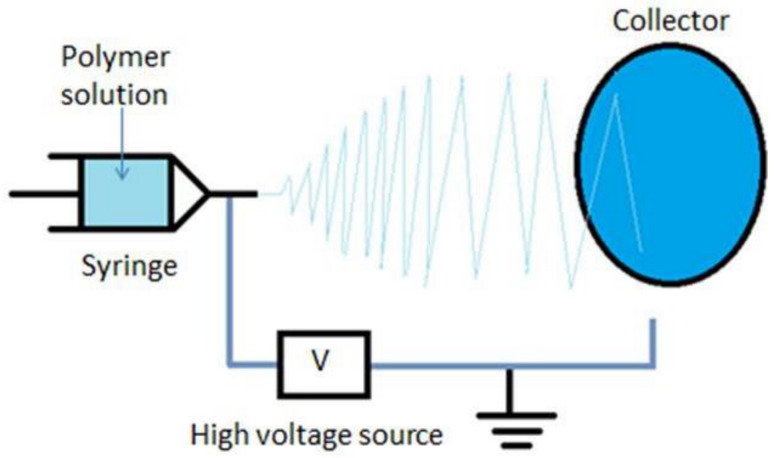
A schematic diagram of the electrospinning setup.

The setup of electrospinning, as shown in Figure 1, includes a high voltage power supply (model CZE1000R, Spellman High Voltage Electronics Corporation, Hauppauge, New York, NY, USA) which is responsible for generating the high electric field strength required for withdrawing the PVA polymer droplets, a syringe pump (NE1000-Single Syringe Pump, New Era, Farmingdale, New York, NY, USA) which is used to regulate the pumping rate of PVA polymer solution, a plastic syringe with 18-gauge metallic needle to store the polymer solution, along with a metallic collector covered with aluminium foil used as a target. The voltage power supply is connected to the needle while the collector is grounded. The distance between the needle tip and the collector is fixed at 15 cm. For the used mixed Ag NPs inside PVA polymer solution, the voltage difference between the needle and target is adjusted to be 25 kV, with a flow rate of the polymer solution at 2 mL/h for 30 min running time per sample. The aforementioned process parameters were found to be optimum for generating the optimum nanofibers mats with minimum beads. 

### 2.3. Nanocomposite Characterizations

The surface morphology of the electrospun PVA nanofibers mats were examined using SEM (JSM-5300, JEOL, Tokyo, Japan). The samples prior to scanning were sputter-coated with gold, the diameters of fibers in randomly selected SEM micrographs were measured using Image-J software. The image of Ag NPs was taken by transmission electron microscopy (JEM-2100F, JEOL, Tokyo, Japan) Thermal stability was investigated through the Thermogravimetric Analyzer (TGA) using a THASS TGA 1000 instrument (KokBiR, Istanbul, Turkey) for the PVA nanofibers embedded with Ag NPs. Samples of 5 mg for each nanocomposite mat were used, and the rate of heating was 10 °C/min. The surface resistance of the Ag NPs-PVA nanocomposite mats was found through I-V characteristics using a Keithley 2450 source meter (Tektronix, Beaverton, OR, USA) along with a manual four probe station (Lucas Signatone, Gilray, CA, USA).

### 2.4. Pulsed Electromagnetic Waves 

A magnetic gun was used to generate magnetic impulses of 50% duty cycle based on an input interrupted current of 80 mA through a Helmholtz coil of 445 turns each and total resistance of 6.8 ohms. The pulsed magnetic waves were directed by the gun to the exposure chamber where the samples were placed at the midpoint between the two sets of Helmholtz coils, 10 cm apart [22,23]. The magnetic field intensity was measured at the point at which samples were placed and confirmed to be 0.32 mT by using a Gauss/Tesla meter model 4048 with probe T-4048.001 (USA) of accuracy ±2%. Moreover, the shape of the magnetic signals was shown on an oscilloscope by using a Linear Hall-effect IC sensor and displayed as square unipolar pulses. The exposure to electromagnetic waves is very challenging and so, the exposure system was adapted under specific experimental conditions. The exposure system was isolated to make sure that the generated magnetic field was not interrupted by any other external nearby electromagnetic fields [24].

### 2.5. Bacterial Strain and Replication 

The bacterial susceptibility test was obtained for two bacterial models namely *Escherichia coli*-ATCC 25922 (*E. coli*) and *Staphylococcus aureus*-ATCC 25923 (*S. aureus*). The bacterial concentration was adjusted to 10^8^ colony forming units (CFU) for each strain to be inoculated in nutrient agar plate (pH of 7.0 ± 0.2) and allowed 24 h incubation at 37 °C. Then, for each strain three colonies were collected from each agar plate and inoculated in 5 mL of sterilized nutrient broth media and allowed to incubate to maintain fresh subcultures. 

### 2.6. Bacterial Availability and Antibacterial Test

The bacterial growing analysis was maintained by using the plate count technique whereas the bacteria cell viability was obtained as percentage relative to the control count as follows: Cell viability% = [average count after treatment/average count of control] × 100. Moreover, the growing kinetics of bacterial growth were obtained by monitoring the optical density (OD at 600 nm) of bacterial solutions every 1 h through a 24 h period of incubation by using the spectrophotometer type (JENWAY 6405 UV/Visible-U.K.). It is worth stating that an OD of 0.1 corresponds to a concentration of 10^8^ cells per cm^3^; the growth density increased in line with the supernatant turbidity increase [25]. In addition, the growth counts were obtained by using the plate count technique for each OD reading to confirm that the turbidity was arising from live bacterial cells. After that, the growth curves for all bacterial samples were plotted of OD readings and incubation time to indicate the growing lag, log, and saturation phases. Furthermore, the growth curves were analyzed and arbitrary growth rate constants were calculated for each bacterial strain under treatment conditions according to the following formula: Arbitrary growth rate constant = 1/t ln(N/N0), where N is the bacterial cell count at time (t) and No the initial cell count [26]. Then, the arbitrary constant values were plotted versus series concentrations of Ag NPs, trend lines were fitted and slope values were calculated to represent the effect of treatment on growing characteristics. In accordance with previous reported data the maximum inhibition effects on *E. coli* and *S. aureus* due to exposure to EMWs, at magnetic field intensity of 0.32 mT for 60 min, were obtained at frequencies of 40 Hz and 20 Hz, respectively [23]. Indeed, bacterial samples from each strain were treated by ENF mats functionalized by series concentrations of Ag NPs (0, 2, 5, 10, and 50 µg/mL) and exposed to EMWs at the previously reported conditions. It is worth clarifying that a supplement of nanofibers mats encapsulated with Ag NPs was adopted by cutting the mats into small pieces of 5 mg weight of each. The cut pieces were separately placed in micro-tubes containing bacterial supernatant of 1 × 10^7^ CFU/mL from each bacterial strain [27].

### 2.7. Bacterial Cytotoxicity Test

Additionally, the cytotoxicity induced by Ag NPs, ENFs, and exposure to EMWs was assessed by monitoring lactate dehydrogenase (LDH), protein, and nucleic acid leakage into the culture medium. The method used to assess the LDH was adapted as published by Kim et al. [28]. On the other hand, the protein contents were performed according to the Bradford method (Bradford) as adapted by Li et al. [29,30] using the Coomassie Protein assay reagent (Pierce, Rockford, IL, United States). LDH and protein levels were measured as the difference in absorbance readings with the microplate spectrophotometer system (Spectramax 190, Molecular Devices, Downingtown, PA, USA) at 490 nm and 595 nm respectively. Finally, the evaluation of nucleic acid leakage was obtained by measuring the amount of acid released at 260 nm as adopted by Reddy et al. [31] and repeated for error minimization [32]. The data obtained from cytotoxicity measurements were represented as percentage values relative to the control samples. 

### 2.8. Statistical Analysis 

The results were represented as mean values, standard deviations, and the significance of differences between data, which were all obtained from One-way Analysis of Variance (ANOVA). The data were statistically significant at “*p*” < 0.05 as defined by the post hoc Tukey test. All statistical analyses were carried out by using the Windows statistical package program (SPSS Inc., Chicago, IL, USA, ver. 21). 

## 3. Results and Discussion

**Figure 2:**
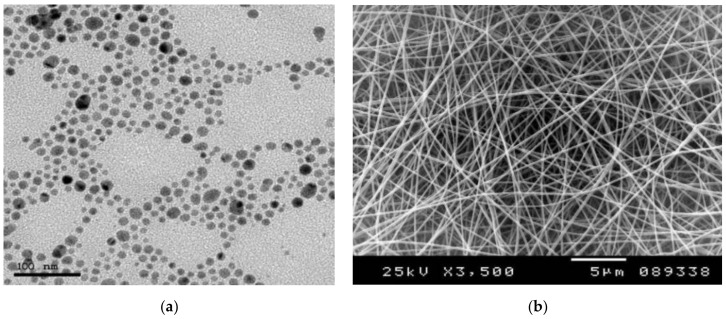
(**a**) TEM image of AgNPs spheres (<20 nm) as previously reported by El-khatib et al. [19], and (**b**) SEM image of electrospun nanocomposite mat.

Figure 2a shows the Transmission Electron Microscope (TEM) image of Ag NPs previously synthesized in our lab and reported in ref. [22], of spherical grain shape along with average size of around 20 nm. Figure 2b shows the SEM image of the formed nanofibers mat, with a mean diameter of nanofibers of less than 100 nm, which offers a better effect of Ag NPs to bacteria due to the higher surface-to-volume ratio. The rise of concerns about bacterial resistance against various types of antibiotics has created an area of research on new effective antibacterial materials.

**Figure 3:**
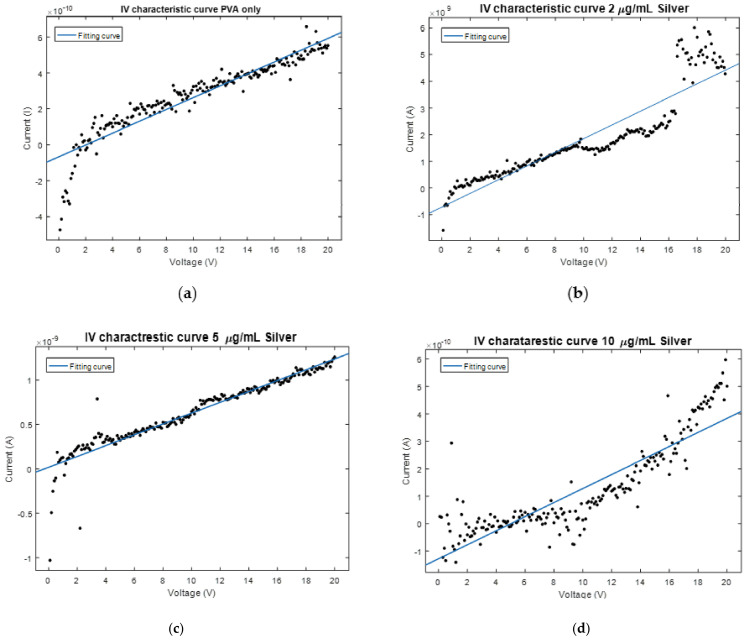
I-V characteristics of Ag NPs-embedded-PVA electrospun nanocomposites at different weight concentration (**a**) 0 μg/mL, (**b**) 2 μg/mL, (**c**) 5 μg/mL, (**d**) 10 μg/mL.

**Figure 4:**
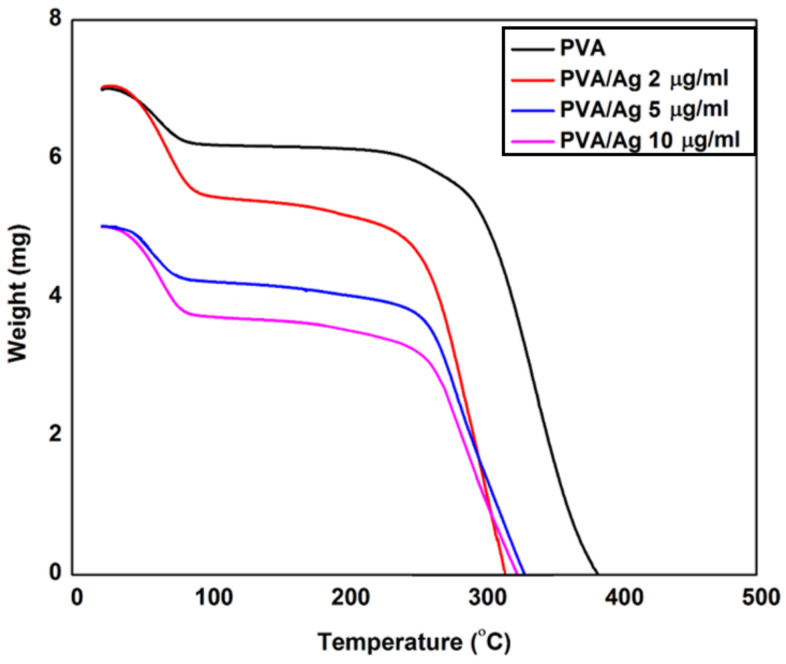
TGA analysis for electrospun PVA nanofibers embedded with different Ag NPs concentrations.

Figure 3 shows the I-V characteristics of our synthesized ENFs of Ag NPs embedded with PVA nanofibers mats at different nanoparticle weight concentrations. The surface resistivity was calculated based on thin-film I-V characteristics equations through four probe analysis [33]. The results showed resistivity decreases of values 194, 123, 84, 51 MΩ/cm^2^ for weight concentration increments of Ag NPs 0, 2, 5, 10 μg/mL, respectively. This demonstrates the incremental existence of Ag NPs on the surface of the nanofibers giving a better possible exposure probability to the bacteria, which is shown later in the anti-bacteria characterizations. Figure 4 shows TGA analysis of the different nanocomposites with increased concentration of Ag NPs. The sample weights remained constant until 270 °C in all nanocomposite samples. Beyond this temperature, the degradation of the weight starts to occur and the rate of degradation slope, increases with increasing weight concentration of silver nanoparticles from 0.055 g/°C at no added Ag NPs up to 0.80 g/°C with added conductive nanoparticles up to 10 μg/mL. This confirms the added distribution of silver nanoparticles inside the electrospun nanofibers with increased thermal conductivity according to the amount of added Ag NPs. 

**Figure 5:**
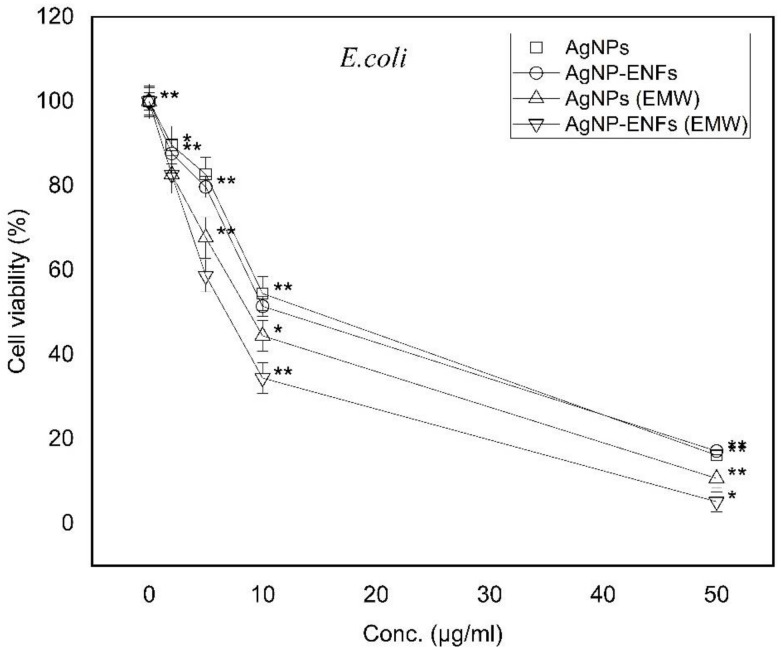
Cell viability percentages of *E. coli* relative to control bacterial counts; * statistically significant, ** statistically highly significant.

**Figure 6:**
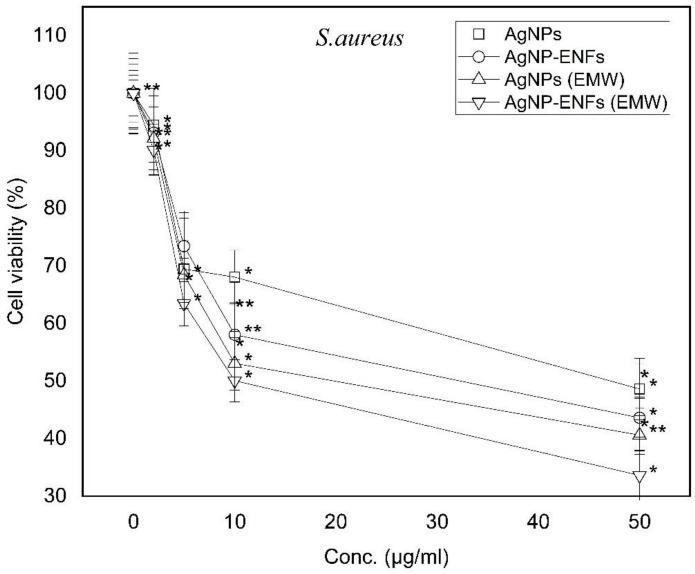
Cell viability percentages of *S. aureus* relative to control bacterial counts; * statistically significant, ** statistically highly significant.

**Figure 7:**
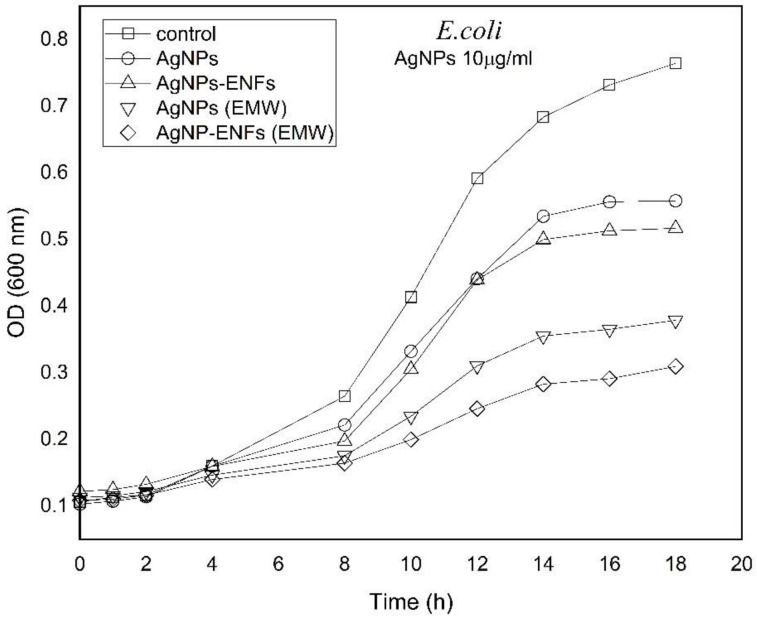
Time-dependent growing characteristic curves of *E. coli* after supplement by (10 µg/mL) Ag NPs alone, Ag NPs-ENFs, and combined with EMWs.

**Figure 8:**
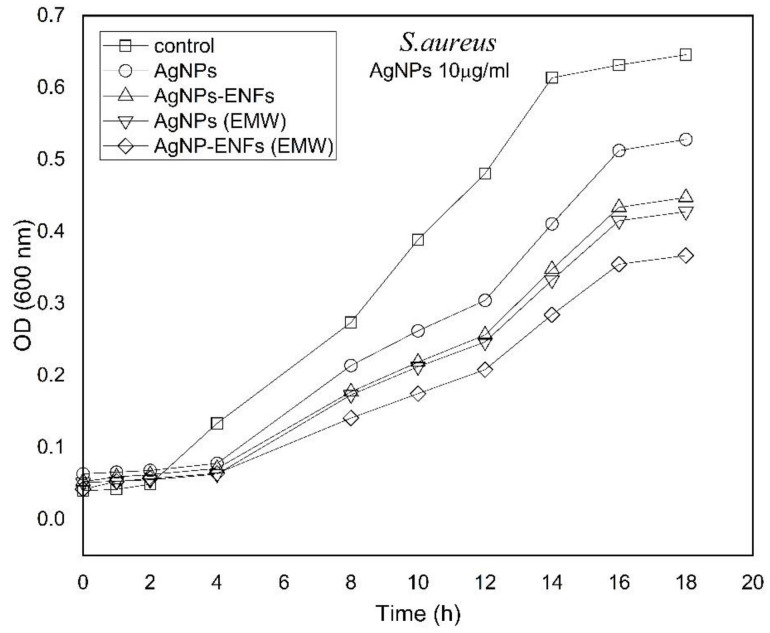
Time-dependent growing characteristic curves of *S. aureus* after supplement by (10 µg/mL) Ag NPs alone, Ag NPs-ENFs, and combined with EMWs.

In our work, the effect of the series of concentrations from Ag NPs (0, 2, 5, 10, and 50 μg/mL) was studied against *E. coli* and *S. aureus* as gram negative and positive bacterial models, respectively. Both Figure 5 and Figure 6 show bacteria cell counts, for both *E. coli* and *S. aureus*, which represent cell viability percentages relative to control bacterial counts. The cell viability for both gram positive and negative bacteria showed clear dependency on the Ag NPs concentration because the inhibition of the bacterial count was found to increase with the incremented concentration of Ag NPs. In comparison to data published elsewhere, the effect of Ag NPs on *E. coli* is greater compared to *S. aureus* and returned to the morphological structure of the cell membrane of each bacterial model [34]. The presence of a thick peptidoglycan layer for *S. aureus* confers protection against Ag NPs attack and prevents its antibacterial impact [35]. The gram positive bacteria have a thicker membrane structure with low ability to be penetrated by the silver ions, whereas the gram negative one has higher susceptibility for ions to be attached at different cellular sites causing cell death. The viability patterns of both bacterial strains showed a similar trend of response towards higher susceptibility to the ENFs and exposure to EMWs. The hybridization of Ag NPs inside ENFs mats has a high volume to surface area ratio and therefore it can cover a larger area of contact with bacterial cell membrane. Similarly, exposure to EMWs has a direct influence on the ionic channel regulations and the concentration gradient across the membrane. The effect of EMWs have pronounced changes on the bacterial preserved packing of the cell membrane whereby the trend of Ag NPs-ENFs on exposure elucidated maximum inhibition of both bacterial strains. Therefore, the effect of EMWs needs to be checked in supporting the nanocomposite mats in the antibacterial process against both *E. coli* and *S. aureus*, as shown in the time-dependent characteristic curves in both Figure 7 and Figure 8.

The growing time-dependent characteristic curves were plottedfor both bacterial strains on application of Ag NPs, ENFs, and EMWs either individually or combined together, as shown in both Figure 7 and Figure 8. The characteristic growth curves were plotted to study the possible kinetic changes that may be attributed to the treatment shown previously in both Figure 5 and Figure 6. The growth curves were selected for Ag NPs (10 µg/mL), because there were no remarkable variations of plateau shape for the plotted curves at other concentrations of Ag NPs for both types of bacteria. The growing curves shown in both Figure 7 and Figure 8 indicate a significant shift in the growth kinetics in being delayed and the curves become shallower with the maximum effect shown to be due to treatment by Ag NPs-ENFs under application of EMWs.

**Figure 9:**
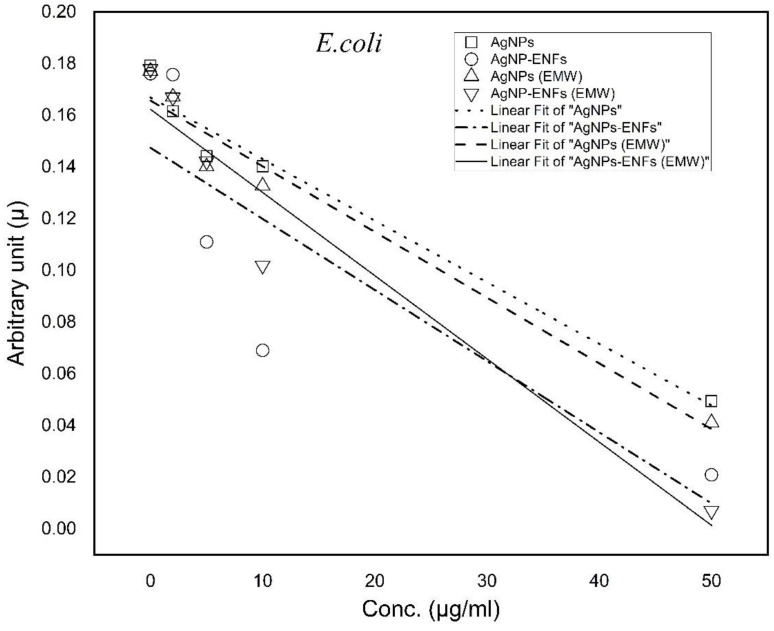
Trend lines of arbitrary constant values for *E. coli* under each treatment condition at different Ag NPs concentrations.

**Figure 10:**
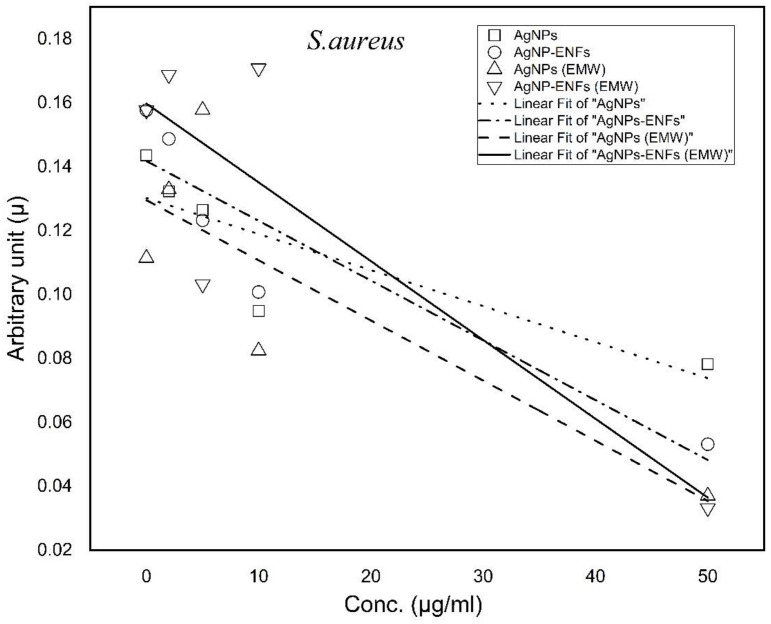
Trend lines of arbitrary constant values for *S. aureus* under each treatment condition at different Ag NPs concentrations.

Furthermore, the growth curves were analyzed in both Figure 9 and Figure 10, along with the calculation of both growth rate constants and arbitrary constants for each treatment condition according to the Ag NPs concentration. The slopes of the arbitrary constant trend lines of the graphs were analyzed and their values tabulated in Table 1. The data indicated that the negativities of slopes have maximum values for samples treated by Ag NPs-ENFs and exposed to EMWs. These findings support the fact that the changes in cell kinetics resulted from delay in the cell production. This can be explained according to alterations in cellular division and abnormality in cell metabolism similar to data reported by Shang L. et al. [36]. Such a synergistic effect of exposure to EMWs and supply of encapsulated Ag NPs inside ENFs elucidated the integrated influence of electromagnetic signals with nanoparticles uptake. This can support the conclusion that EMWs have the ability to interfere directly with the cell membrane signal path of transduction and consequently cause alterations in transmembrane potential [37,38]. Such changes lead to loss of membrane electrochemical balance [39], which in turn enforces ions to cross the membrane through ionic channels without active control [40,41]. The incorrect electrostatic balance of the membrane indicates loss of membrane integrity and reflects dysfunctionality of the cell metabolic processes through cell to cell communication [42,43,44].

**Figure 11:**
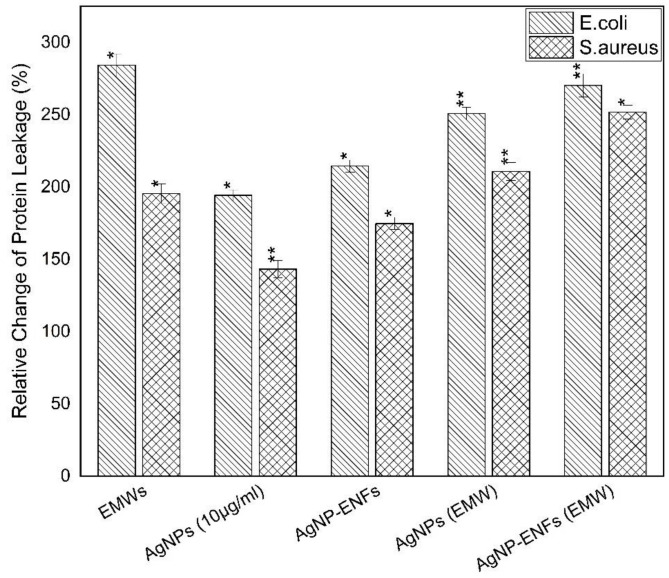
Relative change percentage in protein leakage for *E. coli* and *S. aureus* samples treated by Ag NPs, Ag NPs-ENFs, and EMWs; * statistically significant, ** statistically highly significant.

**Figure 12:**
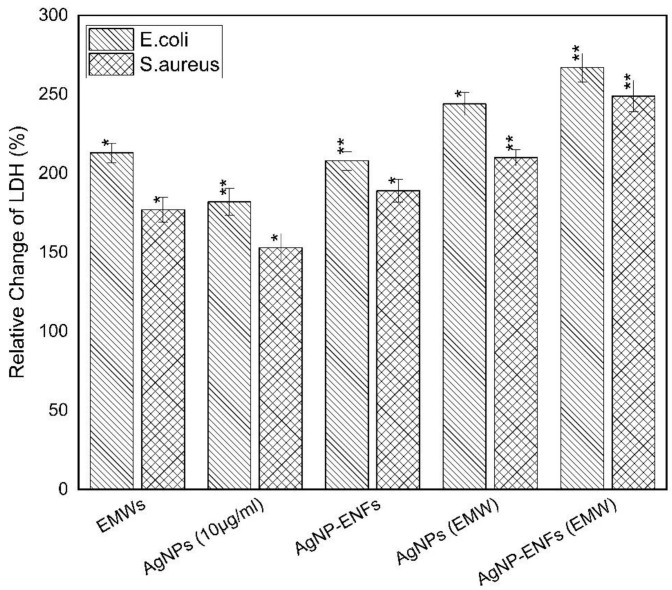
Relative change percentage in LDH for *E. coli* and *S. aureus* samples treated by Ag NPs, Ag NPs-ENFs and EMWs; * statistically significant, ** statistically highly significant.

The former findings of losing cell membrane integrity could be confirmed by measuring the amount of protein and LDH leakage from inside bacterial cells into the extracellular medium [45]. The protein and LDH leakage values were quantified relative to the control samples for both bacterial strains, as shown in both Figure 11 and Figure 12, respectively. By comparing the leakage percentages for all supernatants, it is seen that the release profiles were remarkably elevated over all treated samples. Specifically, the highest levels of leakage were observed for samples exposed to EMWs with supplement of Ag NPs and Ag NPs-ENFs, whether individually or combined together. Therefore, the exposure of EMWs disturbs the membrane integrity by reorientation of its charged macromolecules. Thereby, alterations in the electro-potential energy of the outer membrane exist and consequently more Ag^+^ ions are able to attack the cell at different sites [46].

**Figure 13:**
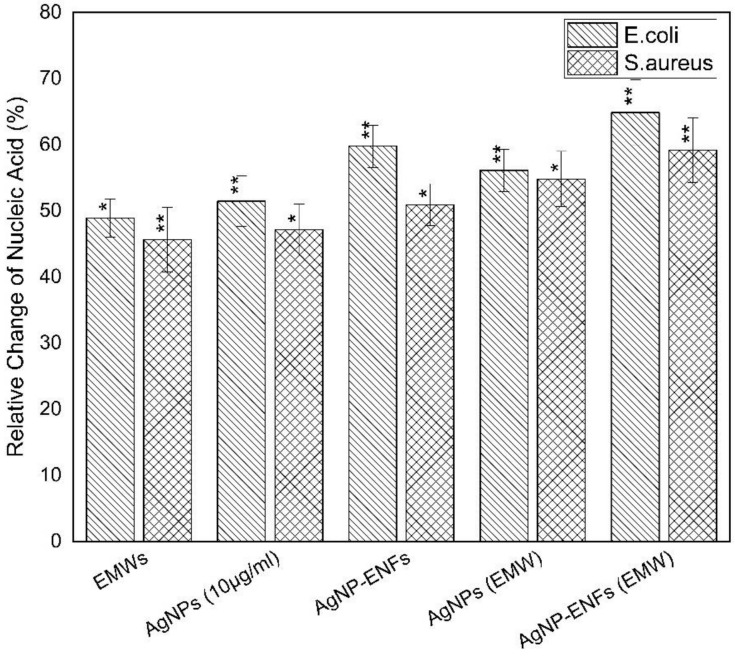
Relative percentage of nucleic acid extortion outside the cell for *E. coli* and *S. aureus* samples treated by Ag NPs, Ag NPs-ENFs and EMWs; * statistically significant, ** statistically highly significant.

As a combination of the previously obtained results, the mutual impact of both the direct contact ability of Ag NPs encapsulated inside the ENFs to the cell membrane surface along with the improper functionality of membrane ionic channels due to EMWs lead to a successful attack against bacterium cells. Thereby, treated bacterial cells showed leakage of cellular materials and extrusion of the cytoplasmic content. Figure 13 shows the percentage of nucleic acid relative to the control samples for *E. coli* and *S. aureus* strains. The obtained data confirmed the previous findings of effective antibacterial performance due to the combination of both Ag NPs-ENFs along with the exposure to EMWs. The increase of leakage of nucleic acid content from inside the cell to outside on EMW exposure indicated the synergistic effect of exposure in adding an external cofactor for killing bacteria at a relatively low concentration of Ag NPs. Also, it is clear that the influence of either Ag NPs or EMWs are highly effective against gram negative bacteria compared to gram positive bacteria. 

## 4. Conclusions

It has been found that the use of nanocomposites of silver nanoparticles (Ag NPs) embedded inside poly (vinyl alcohol) (PVA) nanofibers mats showed a good dispersion of the Ag NPs and consequently a better exposure to bacteria. The functionalized nanomats improved the bactericidal effect of Ag NPs at relatively low concentrations when combined with exposed electromagnetic waves (EMWs). EMWs act as a cofactor for killing bacteria when synergized with nanocomposite mats for maximum bacterial inhibition. Our proposed antibacterial technique proved to be more effective against gram negative bacteria than gram positive bacteria.

## Figures and Tables

**Table 1:** List of negative slope trend line values of arbitrary constants for *E. coli* and *S. aureus* under each treatment condition at different Ag NPs concentrations.

Treatment Condition	Arbitrary Constant (µ) *E. coli*	Arbitrary Constant (µ) *S. aureus*
Ag NPs	−(2.38 × 10^−3^ ± 2.31415 × 10^−4^)	−(1.13 × 10^−3^ ± 3.92243 × 10^−4^)
Ag NPs-ENFs	−(2.75 ×10^−3^ ± 9.99632 × 10^−4^)	−(1.87 × 10^−3^ ± 4.32734 × 10^−4^)
Ag NPs (EMWs)	−(2.54 × 10^−3^ ± 2.77668 × 10^−4^)	−(1.88 × 10^−3^ ± 7.06717 × 10^−4^)
Ag NPs-ENFs (EMWs)	−(3.22 × 10^−3^ ± 4.82432 × 10^−4^)	−(2.47 × 10^−3^ ± 8.13422 × 10^−4^)
